# Intravenous cyclophosphamide pulse therapy in interstitial lung disease associated with systemic sclerosis in a retrospective open-label study: influence of the extent of inflammation on pulmonary function

**DOI:** 10.1007/s10067-018-4171-6

**Published:** 2018-07-10

**Authors:** W. M. T. van den Hombergh, S. O. Simons, E. Teesselink, H. K. A. Knaapen-Hans, F. H. J. van den Hoogen, J. Fransen, M. C. Vonk

**Affiliations:** 1 Department of Rheumatology, Radboud University Medical Centre, Nijmegen, The Netherlands; 2 Department of Respiratory Medicine, Gelre Ziekenhuizen Apeldoorn, Apeldoorn, The Netherlands

**Keywords:** Cyclophosphamide, Interstitial lung disease, Systemic sclerosis

## Abstract

Interstitial lung disease (ILD) is the primary cause of death in patients with systemic sclerosis (SSc). It is thought that chronic inflammation is a key component in SSc-ILD. Treatment, such as cyclophosphamide (CYC), targets this inflammation. We hypothesized that treatment with CYC might be more effective in the inflammatory phase. Therefore, we analyzed whether the extent of inflammation, as assessed by the proportion of ground glass compared to fibrosis, SSc disease duration, the extent of ILD, or baseline diffusion capacity of the lungs (DLCO) < 60%, modifies the effect of intravenous CYC pulse therapy (750 mg/m^2^) on pulmonary function (as measured by FVC, DLCO) in SSc-ILD patients, after 12, 24, and 36 months. Consecutive patients with SSc-ILD receiving CYC pulses between 2003 and 2015 were included. Pulmonary function tests were performed at 0, 6, 12, 24, and 36 months. There were 75 patients included. Forced vital capacity (FVC) (86% of predicted) and DLCO (42% of predicted) were stable after 12, 24 and 36 months of follow-up (*p* > 0.05). Forty-four patients completed 12 cycles of CYC. For the extent of ILD, proportion of ground glass compared to fibrosis, SSc disease duration, and baseline DLCO, there were no differences (all *p* > 0.05) in the course of FVC and DLCO. Treatment with CYC followed by maintenance therapy stabilizes pulmonary function in patients with SSc-ILD over a 3-year period. The extent of ILD, proportion of ground glass, SSc disease duration, and baseline DLCO < 60% did not influence the effect of CYC on pulmonary function.

## Introduction

Systemic sclerosis (SSc) is a generalized auto-immune disease characterized by inflammation, micro-vasculopathy, and fibrosis, affecting skin and internal organs. The primary cause of death in patients with SSc is interstitial lung disease (ILD) [1]. The incidence of ILD in SSc varies from 25 to 90% depending on the method used to identify ILD [2], with a 10-year survival rate in SSc-ILD patients of 70% from onset of SSc-ILD [3]. Although the pathogenesis of SSc-ILD has not been elucidated, it is hypothesized that chronic inflammation of the alveoli leads to progressive lung tissue damage and increasing fibrosis [4–8].

According to EULAR recommendations, cyclophosphamide (CYC) is the first choice of therapy for treating SSc-ILD [9]. Cyclophosphamide acts as a cytotoxic immunosuppressive agent through modulation of lymphocyte function that leads to depression of the inflammatory response and less fibrosis [10, 11]. Several uncontrolled studies showed a positive effect on pulmonary function in patients receiving cyclophosphamide [12–19]. Based on these studies, two double-blind placebo-controlled randomized controlled trials were subsequently carried out [20, 21]. The first, the scleroderma lung study (SLSI), investigated oral CYC and showed a small positive effect on pulmonary function and quality of life after 12 months compared to placebo, which did not sustain after 24 months. The second, the UK lung study, showed a trend towards improvement of forced vital capacity (FVC) after six monthly intravenous (IV) CYC pulses during 12 months compared to placebo. A third study (SLSII) compared CYC to mycophenolate mofetil (MMF) in a randomized controlled setting. Both CYC and MMF were proven to be equally effective in SSc-ILD after 24 months. These results show that CYC can have an effect, albeit small, on pulmonary function in SSc-ILD and quality of life.

The small effect of these studies might be caused by differences in dosage, treatment duration, and administration route as well as a longer SSc disease duration. In the Radboud University Medical Center, patients with SSc-ILD are treated with monthly CYC pulses according to a protocol in which patients are given monthly iv CYC (750 mg/m^2^) for 12 months as soon as the ILD is diagnosed. We hypothesized that the effects of CYC in our cohort of SSc-ILD patients would be equal to or larger than in the aforementioned trials, because of this upfront, high-dose treatment protocol. We also hypothesized that, based on what is known about the mechanism of action of CYC in SSc-ILD [22], CYC may have a larger effect in an “active” inflammatory phase of SSc-ILD than in a later fibrotic phase.

Therefore, in this study, we analyzed whether the extent of inflammation, as assessed by the proportion of ground glass compared to fibrosis, SSc disease duration, the extent of ILD, or baseline diffusion capacity of the lungs (DLCO) < 60%, modifies the effect of intravenous CYC pulse therapy (750 mg/m^2^) on pulmonary function (as measured by FVC, DLCO) in SSc-ILD patients, after 12, 24, and 36 months.

## Methods

### Design

This retrospective study was designed as an observational, long-term cohort including prospective data collection and intention-to-treat analysis. Since 2003, intravenous, monthly CYC pulse therapy is the first of choice therapy for SSc-ILD and/or rapid progressive diffuse SSc at our Department of Rheumatology of the Radboudumc. All patients since then diagnosed with SSc-ILD who started treatment with monthly iv CYC pulses (dose 750 mg/m^2^) were analyzed in this study. Pulmonary function tests (FVC and DLCO) obtained during scheduled follow-up were used to analyze the treatment effect of CYC at 12, 24, and 36 months after start of CYC pulses. Variables that were chosen to reflect the concept of an “active” inflammatory phase of SSc-ILD included the percentage of ground glass, SSc disease duration, extent of ILD by Goh’s classification criteria, and baseline DLCO. These variables were used as inflammatory proxies to analyze the effect on pulmonary function test results over time. Occurrence of adverse events (AE) and survival were also analyzed.

Due to the observational nature of this cohort, no ethical review was needed according to Dutch law and regulations.

### Patients

All patients with SSc-ILD as indicated by high-resolution computed tomography (HRCT) who started intravenous CYC pulse therapy from 2003 in the Radboudumc were included in this study. Patients who received cyclophosphamide for skin involvement were not analyzed in this study. The dosage of CYC was 750 mg/m^2^. Presence of SSc-ILD was defined as presence of ground glass opacity (GGO) or honeycombing or pulmonary fibrosis on HRCT. Patients were followed from the start of CYC treatment up to 3 years. Most patients in our center received mycophenolate mofetil following CYC infusions, during at least 3 years. Before 2013, patients received MMF with an increasing rate; other patients received azathioprine or no follow-up treatment; from 2013 onwards, follow-up treatment with MMF is the standard.

### Assessments

Baseline demographic and clinical data and results from pulmonary function tests (PFTs) and HRCT were collected. Occurrence of the first non-Raynaud (NR) symptom was appointed as disease onset of SSc. Pulmonary function testing was performed at 6, 12, 24, and 36 months after the start of CYC pulses. Spirometry (FVC) and carbon monoxide DLCO were performed according the ATS/ERS guidelines [23]. Predicted normal values were derived from the European Community for Steel and Coal [24].

The presence and extent of GGO and fibrosis on HRCT was assessed according to the criteria by Goh for limited and extensive ILD. HRCT images were scored at five levels: (1) origin of great vessels, (2) main carina, (3) pulmonary venous confluence, (4) halfway between the third and fifth sections, (5) immediately above the right hemi-diaphragm. The total extent of interstitial lung disease was estimated to the nearest 5 % in each of the five sections, with global extent of disease on HRCT computed as the mean of the scores [25].

Two independent physicians were blinded for patient characteristics including PFT results and blinded for purpose of the study performed this assessment, respectively. Cases in which there was disagreement were discussed in a consensus meeting and consensus was reached in every case.

### Outcomes

The primary endpoint in this study was the FVC after 12 months since the first CYC infusion. Secondary outcomes in pulmonary function were DLCO after 12 months, FVC and DLCO after 24 and 36 months, and therapy response according to the American Thoracic Society [26]. A good response to therapy was defined as an increase in FVC of > 10% or an increase in DLCO > 15% compared to baseline (treatment start) [5, 17, 19, 26, 27]. Stabilization was defined as a change in FVC of < 10% and DLCO of < 15%, and a decrease of > 10% in FVC or > 15% in DLCO as worsened SSc-ILD [28]. Further, secondary outcomes were serious adverse events (SAEs) and death related to ILD.

### Effect modifiers

Effect modifiers of interest, reflecting inflammatory activity of ILD by proxy, were the presence of more GGO than fibrosis, SSc disease duration < 3 years, the extent of ILD according to Goh, and DLCO < 60% at baseline. The method of Goh was used to determine whether limited or extensive ILD was present [25]. This staging system consists of a combination of HRCT scan and PFT data and provides discriminatory prognostic information on SSc-ILD [25]. In short, the disease extent on HRCT scan (more or less than 20% of the surface of the lungs) is combined with FVC % predicted (higher or lower than 70%) to stage the ILD as limited or extensive disease [25].

### Statistical analysis

All analyses of FVC and DLCO were conducted on all patients with SSc-ILD starting a first pulse with CYC. Missing outcome data due to dropout were replaced by the last observation carried forward (LOCF), except in the case of death. Per-protocol analysis was also done, including all patients finishing a complete course of 12 pulses [29, 30].

The effect of the predefined variables, chosen to reflect the concept of an “active” inflammatory phase of SSc-ILD, on the effect of CYC on pulmonary function was analyzed. Linear regression analysis was performed with ΔFVC and with ΔDLCO as the dependent variable. For these analyses, potential confounders were regarded as actual confounders if their inclusion led to a change of > 10% of the regression coefficient of the group effect. Analyses were done for every time point: the difference in FVC and DLCO between 0 vs. 12 months, 0 vs. 24 months, and 0 vs. 36 months. All four probable effect modifiers were analyzed separately.

Further, it was explored whether there were baseline differences between responders and poor responders using Student’s *t* test, Mann–Whitney *U* test, chi-square test, or Fisher’s exact test, as appropriate. Occurrence of SAEs was tabulated; survival was analyzed using a Kaplan-Meier plot. A *p* value < 0.05 was deemed to notify statistical significance. Analyses were done using SPSS version 22.0.

## Results

A total of 91 SSc patients were treated with CYC pulses for SSc-ILD; 16 patients were excluded due to the lack of a baseline HRCT (*n* = 15) or PFT (*n* = 1). Baseline characteristics of the 75 eligible patients are summarized in Table 1. Patients with diffuse cutaneous SSc (dcSSc) were more often male with a shorter disease duration. Fibrosis on HRCT was the most prevalent abnormality at baseline and was found in 95% of the patients; GGO was found in 76% of the patients. Both fibrosis and GGO were most often present in the lower lung fields.

**Table 1:** Baseline characteristics

	Total (*n* = 75)	lcSSc (*n* = 33)	dcSSc (*n* = 42)	*P* value
Age in year, mean ± SD	58 ± 11	59 ± 10	57 ± 12	0.45
Male (%)	41 (55)	11 (33)	30 (71)	0.001
Months since first nRP*	20 (8–52)	26 (10–101)	19 (7–37)	0.07
Months SSc diagnosis-CYC*	3 (1–14)	4 (1–20)	3 (1–12)	0.49
SSc duration < 3 years (%)	50 (67)	18 (55)	32 (76)	0.048
Prior MTX use (%)	29 (39)	10 (30)	19 (45)	0.19
ATA (%)	36 (48)	12 (36)	24 (57)	0.074
ACA (%)	6 (8)	6 (18)	0 (0)	0.006
RNP (%)	3 (4)	2 (6)	1 (2)	0.58
ANA (%)	29 (39)	12 (36)	17 (41)	0.72
HRCT				
% ground glass*	9 (1–21)	9 (1–24)	8 (0–19)	0.43
% fibrosis*	11 (6–22)	13 (6–22)	10 (4–24)	0.60
% disease extent*	23 (12–39)	24 (15–41)	23 (10–35)	0.29
GGO > fibrosis (%)	28 (37)	12 (36)	16 (38)	0.88
Limited ILD (%)	33 (44)	13 (39)	20 (48)	0.48
Pulmonary function				
FVC, % of predicted*	86 (72–97)	83 (68–103)	87 (77–95)	0.58
DLCO, % of predicted*	42 (32–56)	42 (33–56)	43 (30–56)	0.87
Baseline DLCO < 60% (%)	59 (79)	26 (87)	33 (85)	0.55

All values are median (p25–75) unless stated otherwise. *SD*, standard deviation; *dcSSc*, diffuse cutaneous systemic sclerosis; *nRP*, first non-Raynaud symptom; *SSc duration < 3 years*, SSc disease duration shorter than 3 years; *CYC*, cyclophosphamide; *MTX*, methotrexate; *ANA*, anti-nuclear antibodies (no ATA, ACA, or RNP); *ATA*, anti-topoisomerase-I antibodies; *ACA*, anti-centromere antibodies; *RNP*, anti-ribonucleoprotein antibodies; *GGO > fibrosis*, more GGO than fibrosis; *FVC*, forced vital capacity; *DLCO*, diffusing capacity of the lung for carbon monoxide; *HRCT*, high-resolution computed tomography

### Course over time

**Fig. 1:**
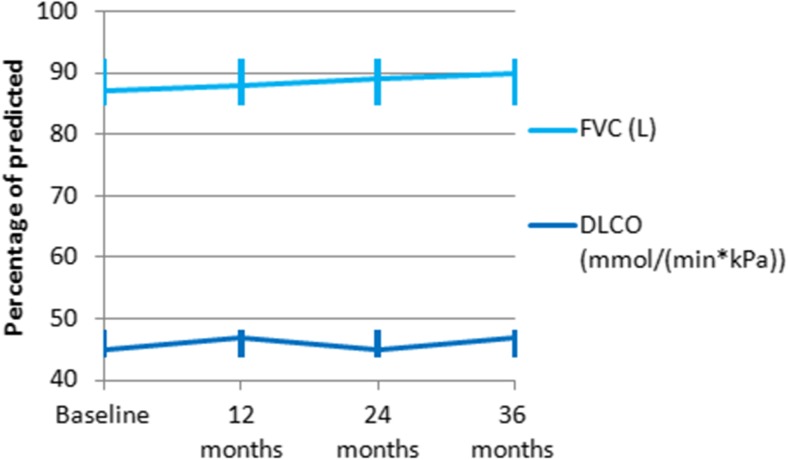
Pulmonary function test results at baseline and during follow-up at 12, 24, and 36 months

PFT results after 12, 24, and 36 months are shown in Fig. 1. Both FVC and DLCO showed a non-significant increase. Table 2 displays the change in FVC and DLCO between baseline and 12, 24, and 36 months, sequentially. Results are shown for the raw data, data adjusted with LOCF and the per-protocol analysis (PPA) of all patients who completed 12 cycles of CYC (*n* = 44). On average, forced vital capacity after 36 months was 1% higher than baseline (95% CI, − 5.5–7.5%). Similarly, there was a non-significant increase of 1.6% (95% CI, − 2.5–5.7) in DLCO after 36 months compared to baseline. Adjusting results for lost-to-follow-up and per-protocol did not lead to significant changes.

**Table 2:** Pulmonary function test during follow-up

		Baseline	Follow-up	Mean difference with 95% CI	*P* value
% Δ baseline vs. 12 months	FVC	86 (72–97)	87 (77–99)	2.0 (−0.7–4.7)	0.15
	FVC LOCF		85 (72–98)	1.2 (−1.5–3.8)	0.38
	FVC PPA		87 (80–101)	1.6 (−1.5–4.6)	0.31
	DLCO	42 (32–56)	45 (36–58)	−0.5 (−2.8–1.7)	0.64
	DLCO LOCF		45 (33–57)	−1.1 (−3.1–1.0)	0.30
	DLCO PPA		47 (37–59)	0.1 (−2.5–2.8)	0.92
% Δ baseline vs. 24 months	FVC	86 (72–97)	91 (70–103)	2.0 (−2.3–6.2)	0.36
	FVC LOCF		89 (70–100)	1.4 (−2.0–4.8)	0.41
	FVC PPA		91 (70–106)	2.6 (−1.1–6.5)	0.17
	DLCO	42 (32–56)	43 (35–57)	0.3 (−3.0–3.5)	0.88
	DLCO LOCF		43 (34–58)	−0.9 (−3.5–1.8)	0.51
	DLCO PPA		48 (37–58)	0.7 (−2.7–4.0)	0.68
% Δ baseline vs. 36 months	FVC	86 (72–97)	90 (72–103)	1.0 (−5.5–7.5)	0.77
	FVC LOCF		89 (70–99)	1.3 (−2.7–5.3)	0.53
	FVC PPA		90 (69–103)	2.2 (−1.9–6.3)	0.29
	DLCO	42 (32–56)	43 (36–61)	1.6 (−2.5–5.7)	0.43
	DLCO LOCF		45 (36–61)	−0.1 (−3.0–2.9)	0.97
	DLCO PPA		48 (37–61)	0.6 (−2.8–4.0)	0.72

PFT values are median (p25-p75). *FVC*, forced vital capacity; *DLCO*, diffusing capacity of the lung for carbon monoxide; *LOCF*, last observation carried forward; *PPA*, per-protocol analysis; *CI*, confidence interval; *p* value of paired samples *t* test baseline versus follow-up (12, 24, or 36 months)

### Effect modifiers

To investigate whether the treatment effect was more pronounced in the early inflammatory phase, we identified four proxies for active, early inflammation: GGO, disease duration, extent of ILD, and baseline DLCO (see “Methods”). In Table 3, “GGO > fibrosis” indicates the difference between GGO > fibrosis and GGO < fibrosis. This is similar for the analyses of the variables “disease duration < 3 years,” “limited vs. extensive ILD,” and “baseline DLCO < 60%.”

**Table 3:** Linear regression analysis

		Δ 0–12 months			Δ 0–24 months			Δ 0–36 months		
		B	SE (B)	*p* value	B	SE (B)	*p* value	B	SE (B)	*p* value
FVC	Constant	− 0.3	4.4	0.96	− 1.1	5.6	0.85	− 2.7	6.5	0.68
	GGO > fibrosis	0.2	2.6	*0.92*	0.9	3.3	*0.78*	1.8	3.8	*0.64*
	Constant	1.5	3.7	0.68	3.0	4.7	0.52	− 2.2	5.4	0.68
	Disease duration < 3 years	− 1.0	2.6	*0.69*	− 2.0	3.3	*0.56*	1.9	3.9	*0.63*
	Constant	1.6	4.2	0.70	3.2	5.3	0.55	3.5	6.1	0.56
	Limited vs. extensive ILD	− 0.9	2.6	*0.72*	− 1.8	3.2	*0.58*	− 2.1	3.7	*0.58*
	Constant	1.3	4.3	0.77	0.6	5.6	0.92	− 2.3	6.5	0.73
	Baseline DLCO < 60%	− 0.4	3.6	*0.91*	0.6	4.7	*0.90*	2.9	5.5	*0.60*
DLCO	Constant	− 0.9	3.5	0.81	0.8	4.4	0.85	− 1.9	4.9	0.70
	GGO > fibrosis	− 0.7	2.1	*0.75*	− 1.6	2.6	*0.54*	0.5	2.9	*0.85*
	Constant	− 3.0	3.1	0.35	− 2.6	3.9	0.51	− 5.5	4.3	0.21
	Disease duration < 3 years	0.8	2.2	*0.73*	0.7	2.8	*0.81*	3.3	3.1	*0.28*
	Constant	− 5.6	3.5	0.12	− 6.1	4.4	0.17	− 4.8	4.9	0.33
	Limited vs. extensive ILD	2.4	2.2	*0.28*	2.9	2.8	*0.30*	2.5	3.1	*0.42*
	Constant	− 1.0	4.8	0.83	5.8	5.9	0.33	4.6	6.6	0.48
	Baseline DLCO < 60%	− 0.7	4.0	*0.86*	− 6.5	4.9	*0.19*	− 5.2	5.5	*0.35*

*FVC*, forced vital capacity; *DLCO*, diffusing capacity of the lung for carbon monoxide; *Δ*, difference in % of predicted between two time points; *B*, beta; *SE*, standard error; *ILD*, interstitial lung disease; *GGO*, ground glass opacity. All values are corrected for baseline values

None of these proxies for early, active inflammation were associated with a higher treatment effect. For example, the change in percentage of predicted FVC values between 0 and 12 months differed 0.2% between GGO > fibrosis and GGO < fibrosis, which was a non-significant difference (*p* = 0.92). The other probable effect modifiers also showed no significant differences in both FVC and DLCO at all follow-up time points. In the per-protocol analysis in the 44 patients who completed 12 cycles of CYC pulse therapy, similar results were seen (not shown).

### Responders

Out of the 75 patients who started treatment with CYC pulses, 14 patients (18.7%) fulfilled the good response criteria during follow-up. A total of 41 patients (54.7%) stabilized and 20 patients (26.7%) worsened during follow-up.

There was a significant difference (*p* = 0.008) between the different response groups in SSc disease duration: the good responders had a median disease duration of 10 months (p25–p75: 4–29); the stabilization group had a median of 25 months (10–98) and in the worsened group the median was 19 (9–34). Baseline FVC was significantly (*p* = 0.04.) lower in the good responders and stabilized patients (median 75 (p25–75: 71–92) and 83 (70–99)) compared to the poor responders (93 (84–113)). A per-protocol analysis in the 44 patients who completed 12 cycles of CYC pulse therapy showed similar results.

### Concomitant medication

A total of 51 patients (68%) received treatment with mycophenolate mofetil during follow-up. There were no baseline differences between the users and non-users of mycophenolate mofetil. Also, there were no differences in FVC and DLCO, nor in the change of FVC and DLCO, during follow-up between patients receiving mycophenolate mofetil and patients not receiving mycophenolate mofetil.

### Treatment discontinuation

A total of 31 patients (41%) withdrew before the completion of 12 monthly cycles of CYC. During the monthly cycles of CYC pulses, four patients died (see section Survival). The reasons for treatment discontinuation in the other 27 patients were progression of ILD (13), pneumonia (3), excessive burden on the patient (2), renal impairment (1), leucopenia/thrombopenia (1), allergic exanthema (1), and leukocytoclastic vasculitis (1). Additionally, there were four patients who completed only six CYC pulses, due to a different choice for the treatment strategy, for example to preserve fertility. In one patient, the discontinuation reason was unclear. There were no significant differences regarding treatment discontinuation between the probable effect modifiers: limited and extensive ILD, more GGO than fibrosis, disease duration, or baseline DLCO < 60%. Although not significant (*p* = 0.35), in 11 out of 59 patients (18.6%) with a baseline DLCO < 60%, the reason for treatment discontinuation was treatment inefficacy, compared to 0 out of 10 patients (0%) who had a baseline DLCO > 60%.

### Adverse events

Adverse events during treatment with CYC occurred in 17 cases (23%): pneumonia (7), anemia (4), leucopenia (2), and hematuria, renal insufficiency, leukocytoclastic vasculitis, and allergic exanthema (1).

No significant differences were seen in occurrence of adverse events between limited and extensive ILD, more GGO than fibrosis, disease duration, or baseline DLCO < 60%.

### Survival

A total of 14 patients died during the CYC pulses (4) or during the 3-year follow-up period (10).

Of the four patients who died during the CYC pulses, one patient died after a complication of vascular surgery, and one died of respiratory insufficiency following congestive heart failure due to progression of ILD and pulmonary hypertension (PH). Two patients died after a probable pneumonia complicating their ILD. There were another 10 patients who died during the 3-year follow-up period: progressive ILD/PH (*n* = 4), progressive ILD (*n* = 2), metastasized colon carcinoma (*n* = 1), sepsis (*n* = 1), acute cardiac asthma (*n* = 1), and SSc-related myocarditis (*n* = 1).

**Fig. 2:**
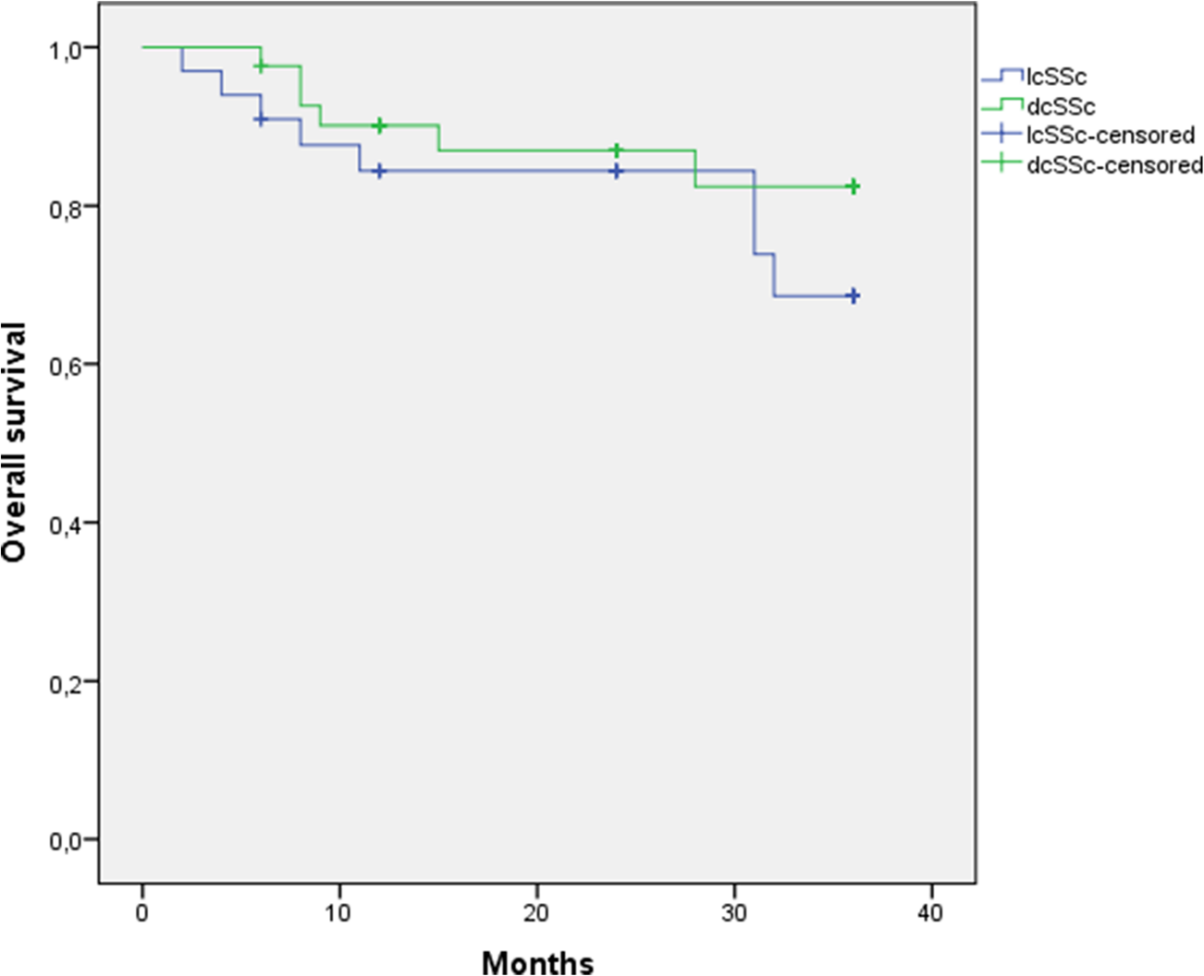
Kaplan–Meier curve. Survival in SSc-ILD treated with monthly CYC pulses

In Fig. 2, the Kaplan–Meier survival curve is displayed, showing that most patients died in the first year after the start of CYC pulses. The overall 3-year survival rate in the 75 patients who started CYC pulses was 81%. Survival in the lcSSc patients was 76% compared to 86% in dcSSc patients; this was a non-significant difference (log-rank 0.30). The effect modifiers (more GGO than fibrosis, disease duration, limited ILD, or baseline DLCO < 60%) showed no significant difference in survival (not shown).

## Discussion

The present study shows that high-dose treatment with CYC leads to a sustained stabilization of pulmonary function in patients with ILD associated with SSc. We found that both vital capacity and carbon monoxide capacity of the lungs remained stable for 3 years after treatment with CYC. The treatment effect seemed to be independent of the inflammatory phase of ILD; proxies for disease activity did not significantly alter the treatment effect.

According to the EULAR treatment recommendations, CYC is the first choice treatment for SSc-ILD [9]. The effect of CYC on pulmonary function as demonstrated in other studies is confirmed by the results of the current study [13–16, 19–21, 31]. In this study, we also were able to show long-term stabilization of pulmonary function during 3 years of follow-up, as well as an overall 3-year survival rate of 81%. Besides, this study has identified a subgroup of good responders. On the opposite, treatment with CYC also has its downsides, with adverse events occurring in almost one in four patients.

As stated in the “Introduction,” we hypothesized that CYC might be more effective if SSc-ILD still has a large inflammatory component while fibrosis is less present. We have collected four proxies of inflammatory activity and inflammation to approach this as closely as possible (the proportion of ground glass, SSc disease duration, extent of ILD according to Goh’s criteria, and baseline DLCO < 60%).

Contrary to our hypothesis, none of these proxies showed could predict a good treatment response in our cohort of SSc-ILD patients. This might have several explanations. First, the treatment effect of CYC might be independent of the extent of ILD. Second, the proxies we chose might have reflected the early inflammatory phase inadequately. Third, inflammation might not be the driving factor for the treatment effect of CYC. Thus, there is a lack of proper proxies for treatment effect in SSc-ILD. The underlying issue is the lack of a gold standard for inflammation in interstitial lung disease. In order to gain further insight, future research should focus on the pathophysiology of SSc-ILD to identify relevant markers that show good respond to CYC treatment.

Predictors of a good response on CYC therapy are pivotal. In our study, 19% had a predefined good response, 55% of the patients had stabilized pulmonary function test results, and 27% worsened during follow-up. Although we could not find a robust treatment effect of our proxies, there was some evidence that patients with a short disease duration respond better. Baseline FVC in the good responders was remarkably significantly lower than that in the poor responder group, but this might be a result of less loss of FVC in the poor responder group: “less loss, less to regain.” The failure of our proxies to identify relevant subgroups of patients that might respond better to cyclophosphamide treatment underlines the need for better treatment surrogates in future trials, especially in light of the rate of serious adverse events seen in the present study.

The use of MMF in SSc-ILD has increased over the last decade. In our center, MMF is often described after CYC infusions. We found no differences in MMF users and non-users in both baseline and follow-up results. Therefore, we could not prove an additional effect of MMF following CYC treatment as was found in the study performed by Tashkin et al. [31].

There are some limitations to this study. The most important limitation probably is our choice of the four surrogate markers for an “early” inflammatory phase in SSc-ILD. There is no gold standard available to measure the amount of inflammation in ILD. We have tried to choose several components which might reflect the early, inflammatory, reversible phase of SSc-ILD. Ground glass opacities seen on HRCT indicate the presence of alveolitis, which is still reversible. Secondly, 75 out of 91 patients who started with CYC pulses because of SSc-ILD were analyzed in this study. The 16 patients all dropped out due to missing baseline HRCT (*n* = 15) or PFT (*n* = 1), not because of specific disease-related causes. Therefore, the probability of bias is small.

In conclusion, overall pulmonary function in SSc-related ILD measured by FVC and DLCO shows stabilization after intravenous CYC pulses during a 3-year follow-up period. One fifth of the patients improved, more than half stabilized, and a quarter worsened. A significant part of the patients died during the follow-up period. The different inflammatory activity proxies, proportion of ground glass, SSc disease duration, extent of ILD, and baseline DLCO do not influence the effect of CYC on pulmonary function. Therefore, based on the results of both previous RCTs and the results of this study, CYC appears to be an appropriate therapy for a large proportion of patients with SSc-ILD; subgroups of patients with good or no response could not be identified. Future studies should focus on identifying relevant markers of inflammation to better select those patients with SSc-ILD that will have the best response to this intensive treatment.
